# Modeling the effectiveness of One Health interventions against the zoonotic hookworm *Ancylostoma ceylanicum*

**DOI:** 10.3389/fmed.2023.1092030

**Published:** 2023-03-07

**Authors:** Martin Walker, Sébastien Lambert, M. Inês Neves, Andrew D. Worsley, Rebecca Traub, Vito Colella

**Affiliations:** ^1^Department of Pathobiology and Populations Sciences, Royal Veterinary College, Hatfield, United Kingdom; ^2^Department of Infectious Disease Epidemiology, London Centre for Neglected Tropical Disease Research, Imperial College London, London, United Kingdom; ^3^IHAP, INRAE, ENVT, Université de Toulouse, Toulouse, France; ^4^Department of Veterinary Biosciences, The University of Melbourne, Melbourne, VIC, Australia

**Keywords:** hookworm, *Ancylostoma ceylanicum*, zoonosis, One Health, intervention, elimination, effectiveness, modeling

## Abstract

Hookworm disease is a major global public health concern, annually affecting 500–700 million of the world's poorest people. The World Health Organization is targeting the elimination of hookworm as a public health problem by 2030 using a strategy of mass drug administration (MDA) to at-risk human populations. However, in Southeast Asia and the Pacific the zoonotic hookworm species, *Ancylostoma ceylanicum*, is endemic in dogs and commonly infects people. This presents a potential impediment to the effectiveness of MDA that targets only humans. Here, we develop a novel multi-host (dog and human) transmission model of *A. ceylanicum* and compare the effectiveness of human-only and “One Health” (human plus dog) MDA strategies under a range of eco-epidemiological assumptions. We show that One Health interventions—targeting both dogs and humans—could suppress prevalence in humans to ≤ 1% by the end of 2030, even with only modest coverage (25–50%) of the animal reservoir. With increasing coverage, One Health interventions may even interrupt transmission. We discuss key unresolved questions on the eco-epidemiology of *A. ceylanicum*, the challenges of delivering MDA to animal reservoirs, and the growing importance of One Health interventions to human public health.

## 1. Introduction

Hookworms are one of the three major soil-transmitted helminths (STHs)—alongside roundworms and whipworms—that collectively affect more than 2 billion people globally, causing significant morbidity (1). Hookworms alone were associated with approximately 1 million disability adjusted life years in 2019, the highest health burden of the three STHs (2). Like *Ascaris lumbricoides* (roundworm) and *Trichuris trichiura* (whipworm), hookworms are targeted by the World Health Organization (WHO) for elimination as a public health problem by 2030 using a strategy of so-called preventive chemotherapy (3). This entails annual or biannual mass administration of anthelmintics to at-risk populations, principally children and women of reproductive age who are at greatest risk of significant morbidity (1).

In Africa and the Americas, hookworm disease is predominantly caused by the anthroponotic species *Necator americanus* and *Ancylostoma duodenale* [but see (4)]. Therefore—in the absence of significant exogenous sources of infection—repeated treatment of human populations at sufficient frequency, coverage and adherence can, in theory, engender elimination (5–7). However, in Southeast Asia and the Pacific, the zoonotic species *Ancylostoma ceylanicum*—which globally infects approximately 100 million people—is the second most common cause of hookworm infection in humans (8–10). Dogs and cats are the main animal hosts of *A. ceylanicum*, and provide a substantial reservoir of infection to humans (11–13). The presence of a large untreated reservoir of infection presents a serious challenge to the elimination of hookworms in these regions and an impediment to reaching the WHO 2030 goals.

Numerous authors have advocated the use of a “One Health” approach to tackle *A. ceylanicum* hookworm (9–11, 14, 15) and indeed the WHO 2030 road map acknowledges the importance of One Health for other NTDs such as rabies, taeniasis, and cystic echinococcosis (1). Such an approach would likely involve expansion of mass drug administration (MDA) to domestic and stray cat and dog populations in settings where *A. ceylanicum* is endemic (15), similar to proposals to tackle zoonotic schistosomiasis in Africa by treating livestock (16, 17) and the existing strategy in China where animals are recognized as key to eliminating *Schistosoma japonicum* (18). Although there exist a number of highly efficacious treatment options for hookworm in cats and dogs—including “spot-on” topical treatments (19–21)—there is currently no empirical evidence on the likely effectiveness of a One Health approach.

In this paper, we develop a novel multi-host transmission dynamics model to compare the effectiveness of a One Health intervention strategy (that targets treatment of both humans and animals) with the current human-only MDA strategy. Motivated by settings in Southeast Asia, where dogs are a major source of *A. ceylanicum* infection (11–13), we consider a range of eco-epidemiological conditions with humans as either “maintenance” hosts—capable of sustaining transmission in the absence of dogs—or non-maintenance, spillover hosts (22). We simulate the impact of MDA starting in 2023 through 2030, aligning to the WHO's elimination timeline (1), quantifying effectiveness in terms of reductions in infection prevalence, and the probability of interrupting transmission.

## 2. Materials and methods

### 2.1. Multi-host transmission dynamics model

Here, we describe the salient features of the multi-host mathematical transmission model developed for this analysis. A complete derivation is given in the Supplementary material section 1.1 and parameter definitions and values are given in Table 1. Briefly, the rate of change in the mean number of hookworms in host *i* at time *t*, *W*_*i*_(*t*), is given by


(1) $$\begin{array}{l} \frac{dW_{i}\left(t\right)}{dt}=\underset{j}{\sum }\left(\mu_{W}+\mu_{j}\right)R{\text{e}}_{i,j}W_{j}\left(t\right)-\left(\mu_{W}+\mu_{i}\right)W_{i}\left(t\right), \end{array}$$

**Table 1:** Parameter definitions and values.

**Parameter**	**Definition**	**Value**	**Reference**
*R*0	Basic reproduction number; average number of adult female *Ancylostoma ceylanicum* produced by a single female worm in the absence of density dependencies	*U*[1, 8]	This work
ω_1, 1_	Proportion of total transmission (*R*0) attributable to host 1 (dogs)	*U*[0.5, 1]	This work
ω_2, 1_	Weighting parameter controlling the proportion of inter-species transmission attributable to host 1 (dogs)	*U*[0.5, 1]	This work
$k_{1}^{*}$	Overdispersion of hookworms among host 1 (dogs) at endemic equilibrium	*U*[0.1, 1]	This work
$k_{2}^{*}$	Overdispersion of hookworms among host 2 (humans) at endemic equilibrium	*U*[0.1, 1]	This work
μ_1_	Per capita mortality rate of host 1 (dogs)	1/5 yr^−1^	
μ_2_	Per capita mortality rate of host 2 (humans)	1/50 yr^−1^	
μ_*W*_	Per capita mortality rate of adult hookworms	2/3 yr^−1^	(23)
μ_*L*_	Per capita mortality rate of hookworm larvae	2 yr^−1^	(23)
*b*	Severity of density-dependent constraints on hookworm fecundity	0.245	(24)
ϵ_1_	Efficacy of spot-on formulation of imidacloprid and moxidectin (Advocate^®^) in host 1 (dogs)	100%	(19)
ϵ_2_	Efficacy of single oral dose aldendazole in host 2 (humans)	90%	(25)
*c*_1_	Coverage of mass drug administration in host 1 (dogs)	25%, 50% and 75%	This work
*c*_2_	Coverage of mass drug administration in host 2 (humans)	75%	(26)

where μ_*W*_ is the per capita mortality rate of adult hookworms (such that 1/μ_*W*_ is the life expectancy; assumed equal in both hosts), μ_*i*_ and μ_*j*_ are the mortality rates of hosts *i* and *j*, respectively, and *R*e_*i, j*_ are components of the effective reproduction number, *R*_e_, describing transmission in (recipient) host *i* from (donor) host *j* (i.e., where *i* = *j* corresponds to transmission between the same host species—“intra-species”—and *i*≠*j* to transmission between different host species—“inter-species”). The components *R*e_*i, j*_ are given by


(2) $$R{\text{e}}_{i,j}=R0_{i,j}\Omega (W_{j}(t),k_{j}(t),b)\Phi (W_{j}(t),k_{j}(t)),$$

where Ω(·) and Φ(·) denote (constraining) density-dependent fecundity (23, 24, 27) and (facilitating) mating probability functions (28, 29), respectively (see Supplementary material section 1.2). Note that parameter *k*_*j*_(*t*)—which (inversely) quantifies the degree of aggregation (overdispersion) of hookworms in host *j* (23)—is dynamic and increases after each treatment round due to imperfect adherence (30) (see Supplementary material section 1.3). The basic reproduction number, *R*0, is given by the dominant eigenvalue of the so-called **K** matrix of intra- and inter-species components, *R*0_*i, j*_ (31, 32). We parameterize matrix **K** as


(3) $$K=\left[\begin{array}{cc} \omega_{1,1}R0 & {\left(\omega_{1,1}\omega_{2,2}R0^{2}\right)}^{\omega_{1,2}}\\ {\left(\omega_{1,1}\omega_{2,2}R0^{2}\right)}^{\omega_{2,1}} & \omega_{2,2}R0 \end{array}\right]$$

where ω_1, 1_ = 1−ω_2, 2_ is the proportion of total transmission (i.e., proportion of *R*0) attributable to host 1 and ω_2, 1_ = 1−ω_1, 2_ controls the proportion of inter-species transmission attributable to host 1 (which is a function of *R*0; see Figure 1).

**Figure 1:**
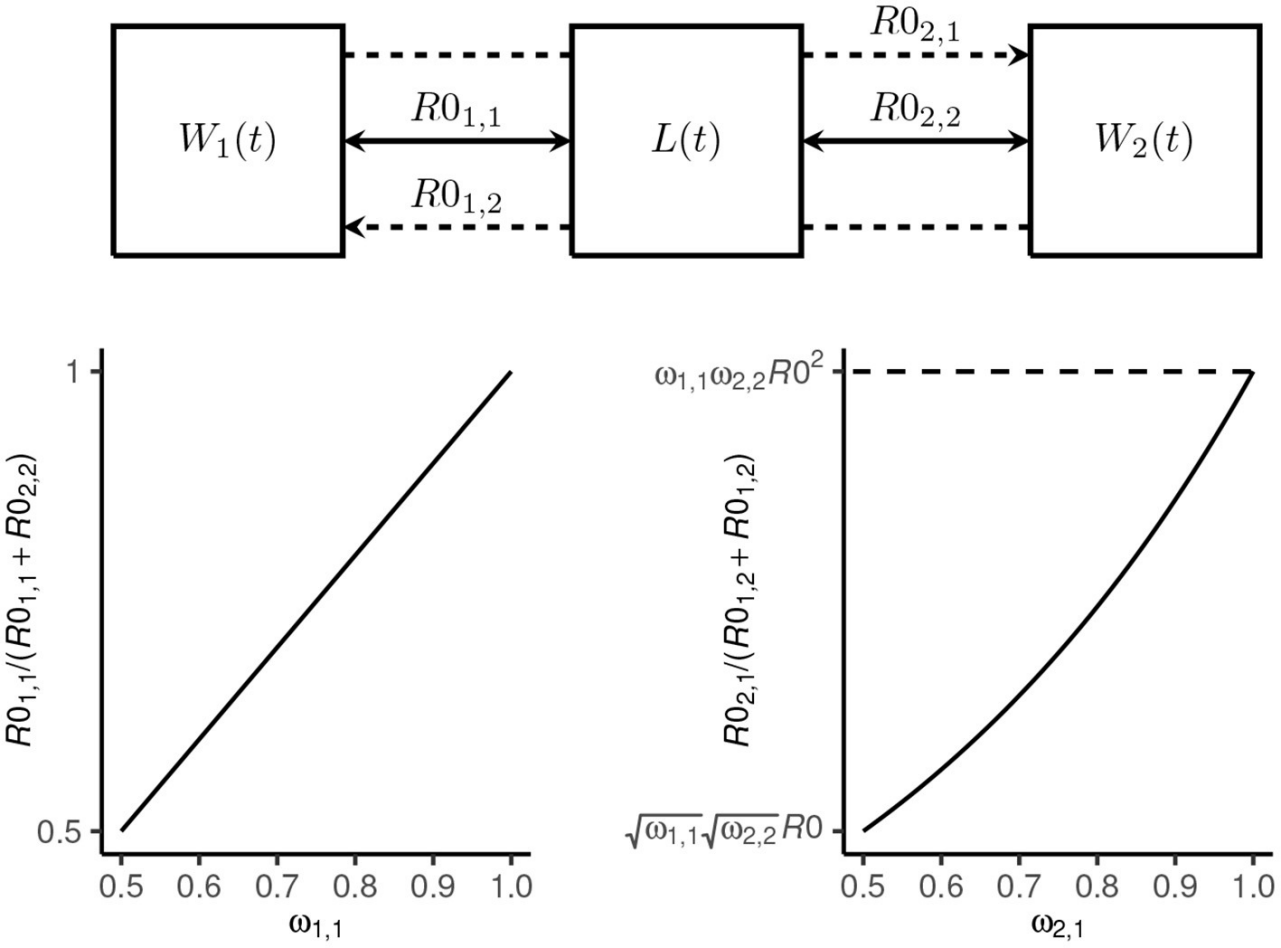
Transmission dynamics model schematic. The mean number of *Ancylostoma ceylanicum* hookworms at time *t* in host 1 (dogs) and host 2 (humans) is denoted *W*_1_(*t*) and *W*_2_(*t*), respectively, and the mean density of larvae in the environment by *L*(*t*). Transmission among and between dogs and humans is coupled by a single shared environment. Intra-species transmission is defined by the reproduction numbers *R*0_1, 1_ and *R*0_2, 2_, respectively (solid arrows). The proportion of total transmission—defined by *R*0—that is attributable to dogs is given by ω_1, 1_ = (1−ω_2, 2_). Inter-species transmission in dogs from humans is defined by *R*0_1, 2_ and in humans from dogs by *R*0_2, 1_ (broken lines). The proportion of inter-species transmission attributable to dogs is proportional to *R*0 for ω_2, 1_ = 1−ω_1, 2_ = 0.5 and proportional to *R*0^2^ for ω_2, 1_ = 1.

### 2.2. Infection prevalence

The prevalence of infection is derived from assuming a negative binomial distribution of hookworms among hosts (23, 33), such that


(4) $$P_{i}(t)=1-{\left(1+W_{i}(t)/k_{i}(t)\right)}^{-k_{i}(t)}.$$

Note that we assume implicitly that prevalence is measured using a perfect diagnostic (i.e., 100% sensitivity and specificity). The “apparent” prevalence would be affected by imperfect sensitivity and specificity, although for PCR, both have been reported as very high (34, 35).

### 2.3. Parameter sampling

We considered *R*0, ω_1, 1_(= 1−ω_2, 2_), ω_2, 1_(= 1−ω_1, 2_), $k_{1}^{*}$, and $k_{2}^{*}$ (where $k_{i}^{*}$ is the degree of overdispersion at endemic equilibrium; see Supplementary material section 1.3) as likely highly variable among different endemic settings. We therefore sampled 10, 000 parameter sets from independent uniform distributions using a Latin hypercube approach (36). We restricted the sampling to settings where *R*0>1 [although note that because hookworms are obligate sexually reproducing parasites, *R*0 = 1 is not a threshold for persistence (37), and therefore some of the parameter sets did not yield stable endemic equilibrium] and where dogs contribute a majority to both total transmission and inter-species transmission (i.e., ω_1, 1_∈[0.5, 1] and ω_2, 1_∈[0.5, 1]; see Figure 1). The overdispersion parameters $k_{i}^{*}$ were sampled from plausible ranges (23, 33) (Table 1).

### 2.4. Modeling interventions

We simulated human-only and human plus dog “One Health” MDA strategies for the 10,000 parameter sets reaching stable endemic equilibrium. For simplicity, we considered only settings without prior intervention (i.e., at endemic equilibrium). We simulated annual MDA for endemic prevalence in humans ≥20% and biannual MDA for prevalence ≥50% (26) starting in 2023 with a final treatment in 2030 (i.e., 8 or 16 annual or biannual rounds, respectively). We assumed that dogs were treated with a spot-on anthelminthic with ϵ_1_ = 100% efficacy (e.g., imidacloprid and moxidectin) (19) and humans with a single oral dose of albendazole, with efficacy ϵ_2_ = 90% (25). Coverage in dogs, *c*_1_, was varied between 25% and 75% and for humans, we assumed a coverage of *c*_2_ = 75%. We modeled treatment as killing instantaneously a proportion *c*_*i*_ϵ_*i*_ of adult hookworms. Note that the model is not age-structured and thus does not capture age-associated variation in infection and transmission and that *c*_2_ = 75% should be considered as a nominally “high” coverage, but is not directly comparable to the 75% target coverage for at-risk groups (children aged 1–14 years and women 15–45 years) set by the WHO (26).

## 3. Results

### 3.1. Endemic settings

**Figure 2:**
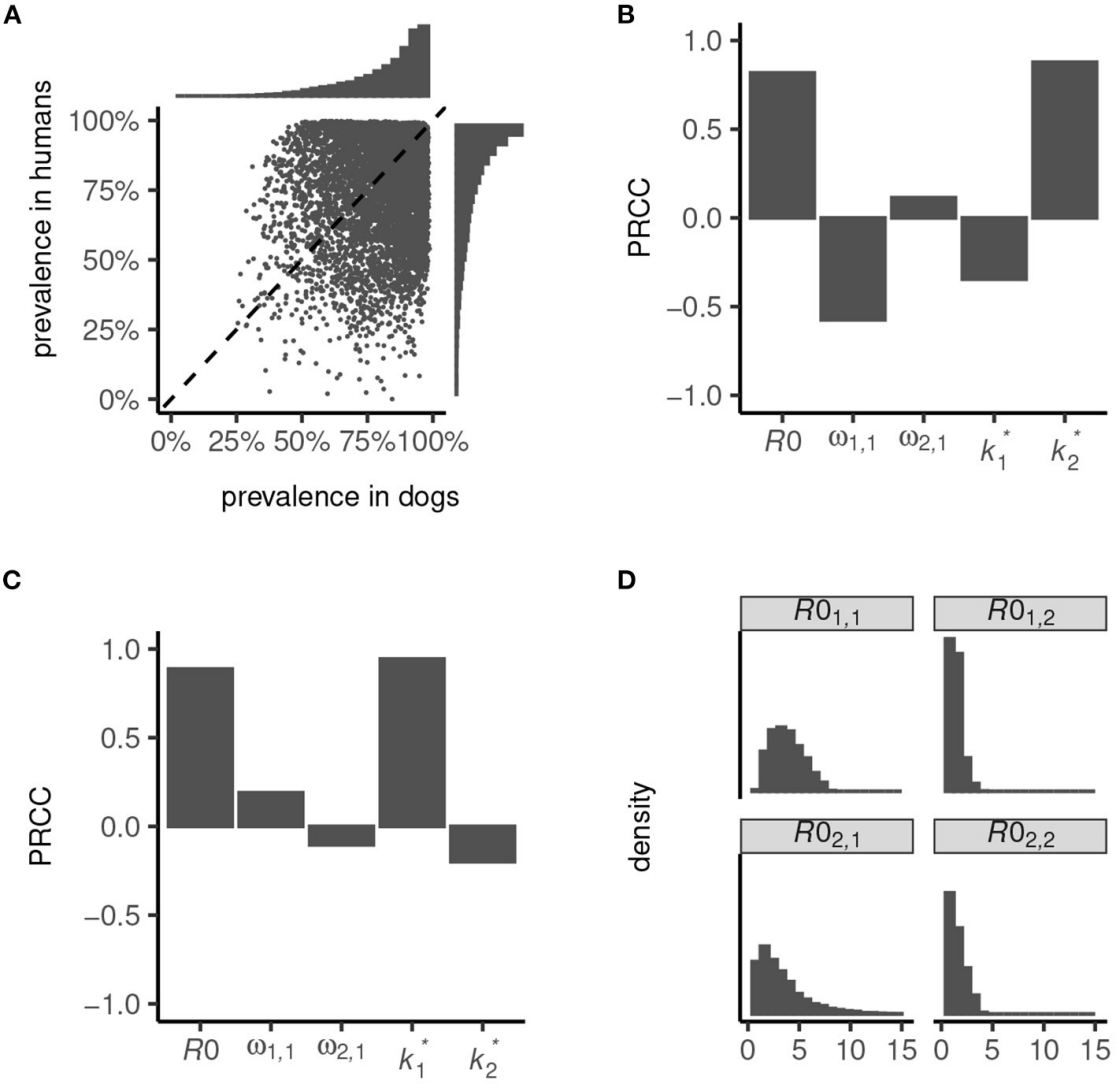
Endemic prevalence of *Ancylostoma ceylanicum* in dogs and humans simulated from the multi-host transmission dynamics model. Variation in prevalence **(A)** was generated by random sampling of parameters governing the basic reproduction number, *R*0, the contribution of dogs to total and inter-species transmission, ω_1, 1_ and ω_2, 1_, and the degree of parasite aggregation among hosts, $k_{1}^{*}$ and $k_{2}^{*}$. The strength of association between infection prevalence in humans **(B)** and dogs **(C)** and parameters *R*0, ω_1, 1_, ω_2, 1_, $k_{1}^{*}$, and $k_{2}^{*}$ is quantified by the partial rank correlation coefficient, PRCC (38). The simulated endemic state is characterized by the distribution of *R*0_1, 1_, *R*0_1, 2_, *R*0_2, 1_, and *R*0_2, 2_ **(D)**.

We used our transmission dynamics model (see section 2.1 and the schematic in Figure 1) to simulate a variety of endemic settings by random sampling of parameters governing the intensity of transmission—defined by the basic reproduction number, *R*0—the contribution of dogs to total and inter-species transmission (determined by parameters ω_1, 1_ and ω_2, 1_) and the degree of parasite aggregation among hosts ($k_{1}^{*}$ and $k_{2}^{*}$; see also Table 1). We considered only settings where dogs are majority contributors to total transmission (i.e., *R*0_1, 1_>*R*0_2, 2_) and inter-species transmission (which is a non-linear function of *R*0; Figure 2), and we restricted *R*0 < 8. These relaxed parameter restrictions resulted in the endemic prevalence in dogs ranging from 26 to 99% and in humans from < 1 to 100% (Figure 2A).

High prevalence in humans is driven principally by a high *R*0, and a low degree of parasite aggregation (higher $k_{2}^{*}$, Figure 2B; see also Section 2.2). Low prevalence is driven by an increased contribution of dogs to transmission (higher ω_1, 1_) and, to a lesser extent, by a decline in parasite aggregation among dogs (higher $k_{1}^{*}$) which lessens the severity of constraints on hookworm fecundity. Like in humans, prevalence in dogs is driven predominantly by *R*0 and the degree of parasite aggregation, $k_{1}^{*}$ (Figure 2C). Less important is their contribution to total and inter-species transmission, ω_1, 1_ and ω_2, 1_, since in all settings dogs are assumed to be majority contributors to *R*0. The degree of parasite aggregation among humans, $k_{2}^{*}$, also has limited impact on prevalence in dogs due to the restrictions imposed on human to dog transmission (i.e., a minimum of 50% of inter-species transmission is assumed to be attributable to dogs; see Figure 1).

Overall, the simulated endemic settings capture a broad array of eco-epidemiological conditions. These range from dogs and humans both as “maintenance hosts”—generally defined by *R*0_1, 1_>1 and *R*0_2, 2_>1 (22, 39), although note that *R*0 = 1 is not a threshold of persistence for dioecious sexually reproducing macroparasites (37)—to dogs as sole maintenance hosts with infection in humans driven by zoonotic “spillover”—generally defined by *R*0_1, 1_>1 and *R*0_2, 2_ < 1 (Figure 2D). In particular, because parameter ω_2, 1_>0.5 permitted a disproportionate contribution of dogs to inter-species transmission (Figure 1), it was possible to capture significant spillover in humans even in low-intensity transmission settings. Hence, certain parameter sets can yield extremely low endemic prevalence in humans (Figure 2A).

### 3.2. Infection dynamics during interventions

We applied the current WHO guidelines on initiating MDA, simulating annual treatment in settings with an endemic prevalence in humans ≥20% and biannual treatment for prevalence ≥50% (26). We modeled only the prevalence of *A. ceylanicum* and therefore, in reality, MDA could be indicated when the prevalence of *A. ceylanicum* is < 20% in settings where other STH species are endemic. We also set the coverage of treatment in humans to the nominal target value of 75% (1) set by the WHO and thus the dynamics elicited by human-only (or the human component of One Health) MDA strategies can be viewed as a “best case” scenario.

**Figure 3:**
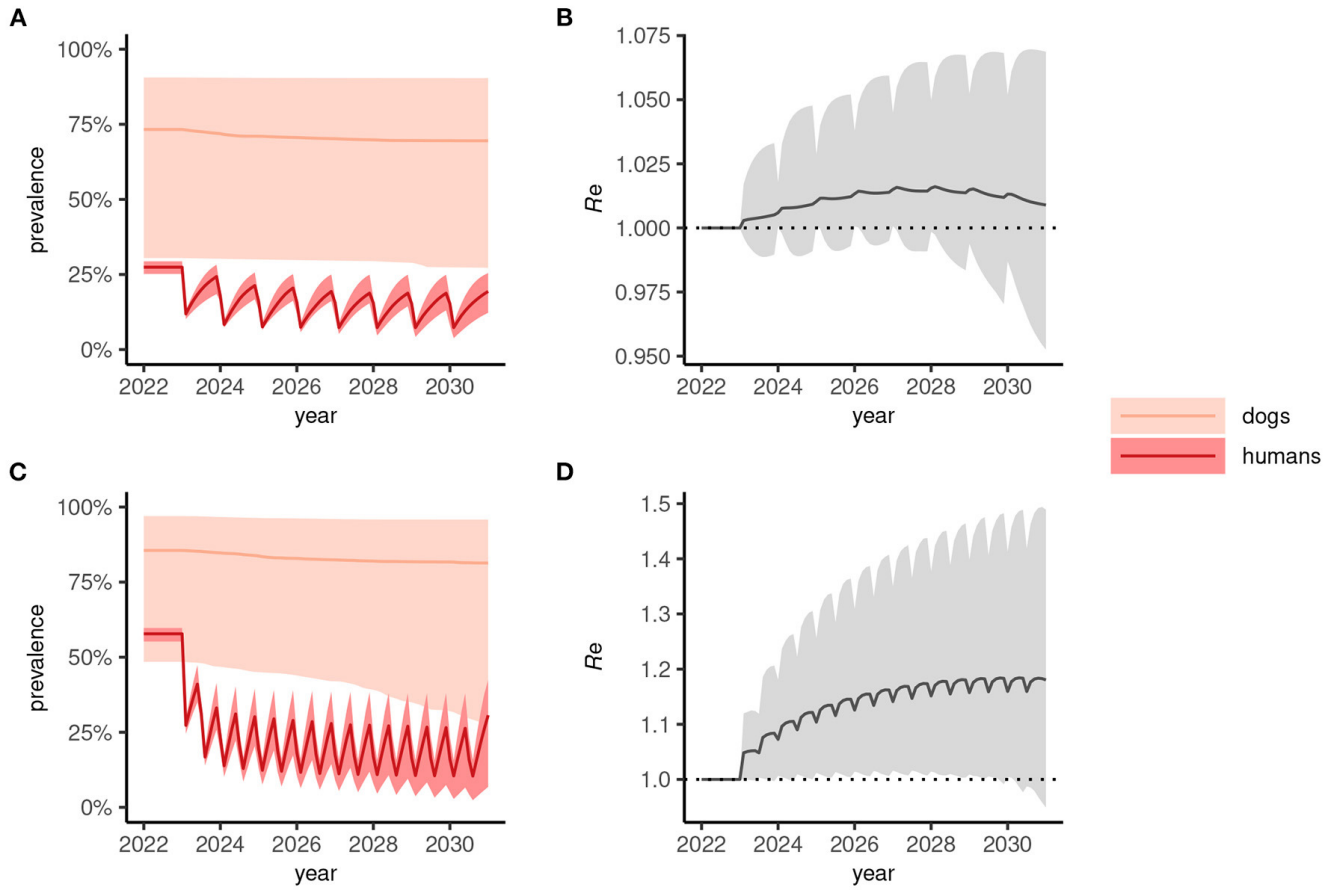
Dynamics of *Anyclostoma ceylanicum* infection prevalence during 8 years of human-only mass drug administration (MDA) simulated using the multi-host transmission dynamics model. For illustration, parameter sets were selected that gave an endemic prevalence in humans of 25–30% **(A)** and 60–65% **(C)**. The solid colored lines indicate the median prevalence of infection (from the sampled parameter sets) in dogs and humans (as indicated) during annual **(A)** or biannual **(C)** MDA at 75% coverage and the lighter shaded areas indicate the extent of the 5th and 95th percentiles. The associated effective reproduction numbers, *R*_e_, are shown in **(B)** and **(D)**, the solid line indicating the median and the shaded gray area the 5th and 95th percentiles. Below the threshold *R*_e_ = 1, transmission is interrupted and the parasite population tends to elimination.

In human-only treatment strategies, the prevalence dynamics often reach a “pseudo-equilibrium,” whereby transmission is suppressed but relatively stable for both annual (Figure 3A) and biannual (Figure 3C) MDA. In these circumstances—where the effective reproduction number, *R*_e_>1 (Figures 3B, D)—stopping MDA would result in resurgence.

**Figure 4:**
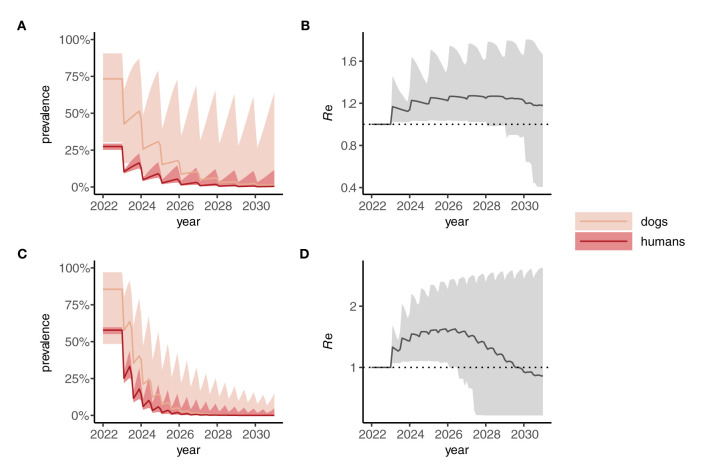
Dynamics of *Anyclostoma ceylanicum* infection prevalence during 8 years of “One Health” (dog plus human) mass drug administration (MDA) simulated using the multi-host transmission dynamics model. For illustration, parameter sets were selected that gave an endemic prevalence in humans of 25–30% **(A)** and 60–5% (C). The solid colored lines indicate the median prevalence of infection (from the sampled parameter sets) in dogs and humans (as indicated) during annual **(A)** or biannual **(C)** MDA at 75% coverage in humans and 50% cover in dogs. The lighter shaded areas indicate the extent of the 5th and 95th percentiles. The associated effective reproduction numbers, *R*_e_, are shown in **(B)** and **(D)**, the solid line indicating the median and the shaded gray area the 5th and 95th percentiles. Below the threshold *R*_e_ = 1, transmission is interrupted and the parasite population tends to elimination.

When a One Health strategy is implemented and MDA is extended to include dogs (assumed to be delivered at the same frequency as indicated for humans), prevalence in both dogs and humans declines progressively toward 2030 (Figures 4A, C) and, on average, *R*_e_ is suppressed below 1 (Figures 4B, D) indicating interruption of transmission. Note that scenarios in which only dogs are treated were not considered here but would likely—depending on the intensity of intra- and inter-species transmission—suppress prevalence more rapidly in humans than seen in dogs for the corresponding human-only treatment strategies shown in Figure 3.

### 3.3. Effectiveness of One Health interventions

**Figure 5:**
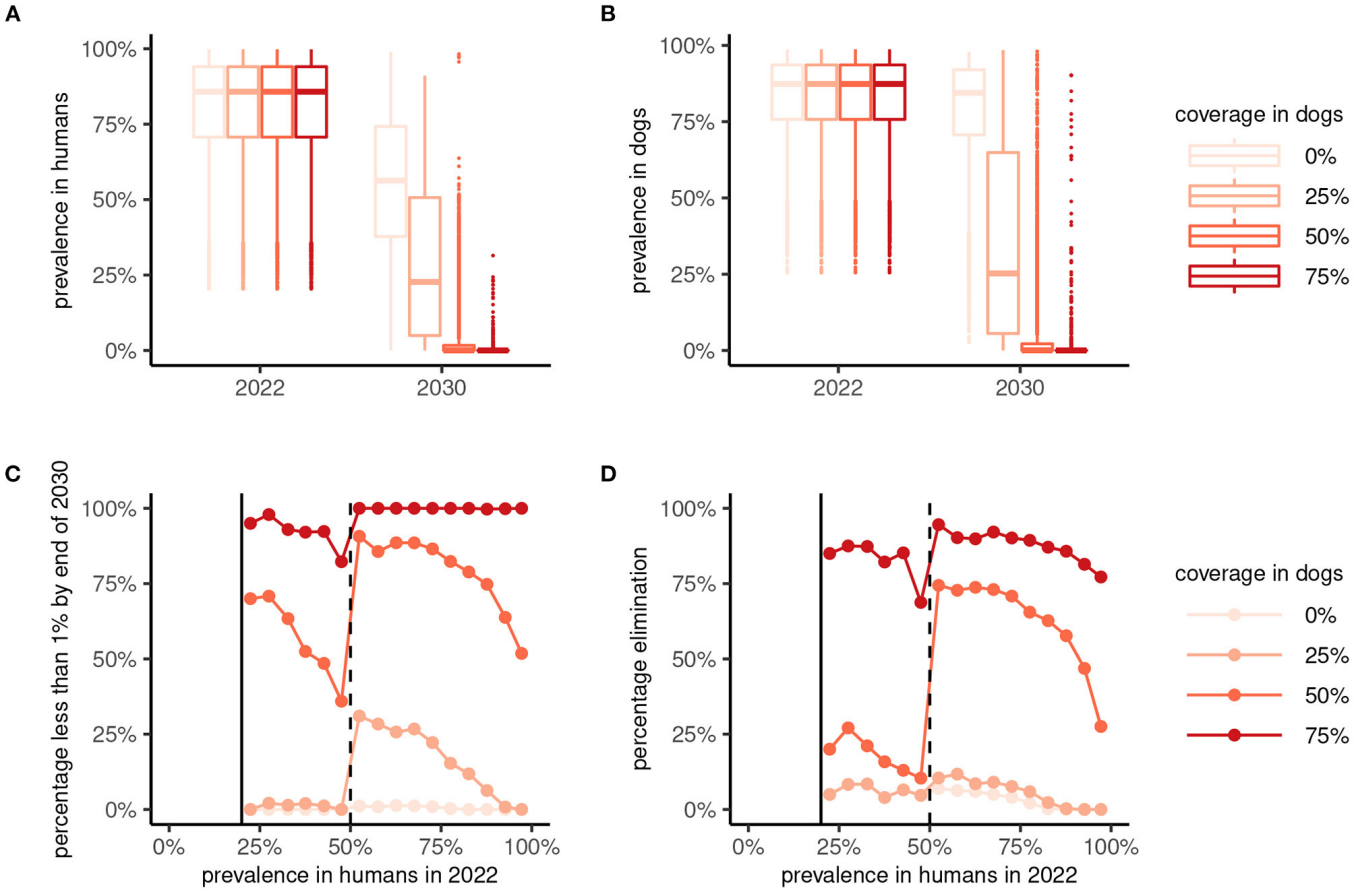
Effectiveness of 8 years of human-only or “One Health” (human plus dog) mass drug administration (MDA) against *Ancylostoma ceylanicum*. Boxplots show the prevalence of *A. ceylanicum* in humans **(A)** and dogs **(B)** at endemic equilibrium in 2022 and after 8 years of MDA at the end of 2030. Human-only MDA is indicated by a coverage in dogs of 0% (lightest color), with the coverage in dogs for One Health MDA varied between 25% and 75% as indicated. The bottom panels show the percentage of parameter sets yielding a prevalence < 1% in humans **(C)** or interruption of transmission [elimination; *R*_e_ < 1; **(D)**] by 2030 against the endemic prevalence in 2022. MDA was not simulated for an endemic prevalence in humans of < 20%.

The implementation of a One Health MDA strategy has a significantly greater effect on suppressing the prevalence of *A. ceylanicum* hookworm in humans (and in dogs) by the end of 2030 compared to human-only MDA (Figures 5A, B). In particular, reaching a coverage of more than 50% in dogs suppresses the prevalence in humans to below 1% in a majority of scenarios (Figure 5C). Moreover, reaching 75% coverage in dogs yielded high (≥75%) chances of interrupting transmission and achieving local elimination (Figure 5D).

## 4. Discussion

*Ancylostoma ceylanicum* is the second most common cause of hookworm infection in Southeast Asia and the Pacific (8–10), with substantial reservoirs of infection in dogs and cats (11, 12). Here, we have modeled the potential effectiveness of a One health intervention strategy that expands MDA beyond only humans to also target the zoonotic reservoir of infection. Reflecting the uncertain epidemiology of *A. ceylanicum*, we simulated a broad range of plausible eco-epidemiological settings involving a single animal reservoir (here, dogs, which are major source of *A. ceylanicum* infection in some regions of Southeast Asia). We show that even modest MDA coverage (between 25% and 50%, assuming perfect adherence and drug efficacy) of the animal host may substantially improve effectiveness compared to current human-only strategies. Even in highly endemic treatment-naive settings, 50% MDA coverage is likely to suppress the prevalence of *A. ceylanicum* to < 1% by 2030 and may also achieve local elimination of transmission.

Our results indicate that One Health interventions will be essential to reaching the WHO 2030 elimination goals (1) in many settings where *A. ceylanicum* is endemic (9–11, 14, 15). It is noteworthy that the current WHO elimination goals do not mention *A. ceylanicum* (rather, just the the two most globally common species *A. duodenale* and *N. americanus*) (1). This may reflect a recognition of the likely limitations of human-only MDA where *A. ceylanicum* is endemic or a simple oversight of the emerging importance of zoonotic hookworm in the Asia-Pacific region. Irrespective, our results show clearly that—even with optimistic assumptions of human MDA coverage—the prevalence of *A. ceylanicum* hookworm may remain stubbornly high by 2030 without a One Health approach.

Intuitively, it is unsurprising that where *A. ceylanicum* is endemic—and animals (dogs) are the dominant driver of transmission, as modeled here—targeting only humans is unlikely to be an effective intervention. Likewise, for hookworm in general, it is unsurprising that treating only school-age children is a sub-optimal strategy because of the typical age-infection profiles that continue to increase into adulthood (40). Modeling provides a formal quantitative framework with which to compare these assertions with alternatives [such as treating humans and animals, or treating whole communities rather than only school-age children (41–43)]. Hence, it is important that our results are viewed as illustrating the need and the potential effectiveness of One Health interventions, not as predictions. Indeed, we have deliberately used a highly simplified modeling framework (e.g., host demographic structure is omitted; only a single animal reservoir is considered) both to enhance analytical tractability and to avoid over-interpretation of the results.

There currently remains too much of the eco-epidemiology of *A. ceylanicum* that is unknown to entertain more predictive modeling approaches. In particular, we are unaware of any studies that have attempted to quantify the relative contribution of humans and animal hosts to transmission [such as those for zoonotic schistosomiasis (44, 45)], although the few studies collecting data from humans and animals in the same epidemiological setting suggest that human-to-human transmission is likely substantially less common than animal-to-human transmission (11, 12). Moreover, estimates of infection intensity—critical to understanding intra- and inter-species dynamics—have only relatively recently been developed, using quantitative PCR techniques that can be calibrated to more traditional parasitological measures (i.e., egg counts) to assist with interpretation (46–49), including the calculation of drug efficacy (15). Indeed, we could not model egg counts here [or the prevalence of “moderate” or “high” intensity infection (1, 26)] because of these unknowns.

The inability to model explicitly quantities relating to the elimination of hookworm as a public health problem [i.e, prevalence of “moderate” or “heavy” infections < 2% (1)] meant that we could not evaluate the likelihood of One Health MDA strategies reaching this goal. Nevertheless—and irrespective of whether this goal is feasible where *A. ceylanicum* is endemic—resurgence is a risk in any settings where transmission is not interrupted (50, 51). The challenge posed by untreated zoonotic reservoirs is analogous to the question of whether treating only at-risk human population groups (i.e., children and women of childbearing age) will be sufficient to achieve sustained elimination of STHs more generally (41, 42). While treating only at-risk groups may be sufficient to drive prevalence to very low levels by 2030, modeling has shown that stopping intervention will risk resurgence in many endemic settings (50, 51). Hence, it has been argued that without wider community coverage aimed at breaking transmission (6, 7, 42), sustained elimination could require continuation of intervention (i.e., MDA) almost indefinitely. Our results concord with this assertion; without One Health interventions that go beyond human hosts, sustained elimination of *A. ceylanicum* is unlikely.

The use of modeling to demonstrate the potential effectiveness of One Health interventions where *A. ceylanicum* is endemic is, of course, much easier than the myriad complexities associated with implementation. First, while being most common in Southeast Asia and the Pacific, the geographic extent of *A. ceylanicum* continues to expand with the increasing use of molecular methods that distinguish hookworm species. Indeed, *A. ceylanicum* has recently been identified for the first time in the Americas (52, 53). Second, diagnosis and geographical mapping relies on molecular PCR methods which are more costly and resource-intensive than traditional parasitological methods. Third, although topical (spot-on) anthelminthic formulations—which would be a highly practicable mode of treatment—are highly efficacious (19–21), they are also expensive, intended for the commercial market of pet owners. Hence, the implementation of MDA in impoverished communities would require low-cost procurement or donation.

It is also important to reiterate that the modeling presented here considers only settings where there is a single animal reservoir of infection that is relatively straightforward to target for intervention (MDA). Yet *A. ceylanicum* can be common among both dogs and cats (12). Consequently, the implementation of an effective One Health intervention would require identification of the main animal reservoirs of infection that contribute substantially to human infection. This would incur further resource overheads and logistical complexity to implementation. Indeed, if in our modeling framework we included both dogs and cats as equally contributing to infection in humans, but only the former were targeted for MDA, the effectiveness (and likely also the cost-effectiveness) of the intervention in reducing human infection would be greatly diminished. However, in a recent study performed across eight countries in Asia, hookworms were significantly higher in dogs than in cats, with implementation of educational programs deemed crucial for the control of zoonotic infections of companion animals in Asia (54). While MDA is the cornerstone of the WHO's strategy to eliminating STHs, other approaches such as improved water and sanitation (WASH), education and awareness, infrastructure development, and food safety are also advocated as complementary activities (1). Additionally, treatment of animals, vaccination, and animal husbandry and management practices (all under a One Health umbrella) are also specifically supported by the WHO in the context of other NTDs such as rabies, taeniasis, and echinococcosis. Hence, while the implementation of a One Health approach to tackle *A. ceylanicum* hookworm would undoubtedly present challenges, similar approaches are not without precedent for other NTDs.

## 5. Conclusions

Although *A. ceylanicum* has been recognized as a multi-host parasite since 1913, it has only been in the past decade that it has been recognized as of significant public health importance (4). This period has seen a great expansion in the use of molecular approaches that have blurred the lines of host specificity in other helminth species, including schistosomes (44, 55), and other STHs (56, 57). It is thus unquestionable that One Health approaches to tackling these neglected diseases will be increasingly emphasized as inroads are made into the human reservoirs of infection while animal reservoirs remain largely unchecked. We have shown that a One Health MDA strategy could be highly effective against *A. ceylanicum* hookworm in endemic regions of Southeast Asia and beyond and will be essential for sustained elimination and reaching the WHO 2030 goals. While there remain challenges to implementation—as well as significant gaps in knowledge on the eco-epidemiology of zoonotic hookworms—this work illustrates the potentially substantial impact of a One Health approach to improving human public health.

## Data availability statement

The datasets presented in this study can be found in online repositories. The names of the repository/repositories and accession number(s) can be found below: The model code used for this analysis is publicly available at https://github.com/martwalker/zoonotic-hookworm.

## Author contributions

MW, SL, and VC conceptualized the work. MW conducted the analysis and drafted the manuscript. MW, SL, and MN built the model. MW, SL, RT, and VC edited the manuscript. All authors contributed to the article and approved the submitted version.
