# Supplementary material for: Modeling the effectiveness of One Health interventions against the zoonotic hookworm Ancylostoma ceylanicum
Source: Front Med (Lausanne). 2023 Mar 7;10:1092030. doi: 10.3389/fmed.2023.1092030 (PMC10028197; doi:10.3389/fmed.2023.1092030)
Supplement: Supplementary file 1 [file Data_Sheet_1.PDF]

Supplementary Material

1 MATERIALS AND METHODS

1.1 Transmission model derivation

1.1.1 Reproduction numbers

We start by defining the intra- and inter-host reproduction numbers, $R0_{ij}$ as the product of the average number of female worms in (recipient) host i produced by a single infectious larva in the environment $R_{E\to i}$ and the average number of larvae in the environment produced by a single adult female worm in (donor) host j (in the absence of density-dependent constraints), $R_{j\to E}$,

$$R0_{i,j} = R_{E\to i}R_{j\to E}. \tag{S1}$$

The underlying parameters defining $R_{E\to i}$ and $R_{j\to E}$ relate to the effective contact rate of hosts with infectious larvae and the deposition rate of eggs into the environment (Anderson et al., 2014; Anderson, 1985). Both processes will likely be highly variable between hosts (e.g. through behavioural differences in faecal contamination of the environment) and among settings (e.g., for humans, the availability and use of sanitation facilities and the use of footwear) and are not necessarily symmetrical (e.g., humans may frequently contaminate the environment but may be protected from infection through the use of shoes). There may also be biologically mediated differences in $R_{E\to i}$ depending on the infectiousness of *Ancylostoma ceylanicum* to different hosts.

For a two-host model (i.e., humans and dogs), the basic reproduction number $R0$ is given by the dominant eigenvalue of the so-called $\mathbf{K}$ matrix (Diekmann et al., 1990; Dobson, 2004)

$$\mathbf{K} = \begin{bmatrix} R0_{1,1} & R0_{1,2} \\ R0_{2,1} & R0_{2,2} \end{bmatrix} \tag{S2}$$

which is found by taking the determinant of $\mathbf{K} - \lambda\mathbf{I}$ to yield the characteristic polynomial and solving for the largest absolute value of $\lambda = 0$,

$$\lambda^2 - (R0_{1,1} + R0_{2,2})\,\lambda + R0_{1,1}R0_{2,2} - R0_{1,2}R0_{2,1} = 0. \tag{S3}$$

Since from Equation S1 $R0_{1,2}R0_{2,1} = R0_{1,1}R0_{2,2}$—which arises from the assumption of hosts interacting with a single environmental source of infectious larvae (Fenton et al., 2015)—it follows that in this two-host single-environment case,

$$R0 = R0_{1,1} + R0_{2,2}. \tag{S4}$$

By defining $\omega_{1,1}$ as the proportion of total transmission (i.e., $R0$) contributed by host 1, it follows that

$$R0_{1,1} = \omega_{1,1} R0 \tag{S5}$$

and

$$R0_{2,2} = \omega_{2,2} R0 \tag{S6}$$

where $\omega_{2,2} = 1 - \omega_{1,1}$. Because $R0_{1,2}R0_{2,1} = R0_{1,1}R0_{2,2}$, we can now write

$$R0_{1,2}R0_{2,1} = \omega_{1,1}\omega_{2,2}R0^2. \tag{S7}$$

By defining $\omega_{2,1} = 1 - \omega_{1,2}$ as a weighting constant that controls the proportion of inter-host transmission attributable to host 1—and noting that $R0_{1,2}$ and $R0_{2,1}$ combine multiplicatively as a function of $R0$—we can further define

$$R0_{1,2} = \left(\omega_{1,1}\omega_{2,2}R0^2\right)^{\omega_{1,2}}, \tag{S8}$$

and

$$R0_{2,1} = \left(\omega_{1,1}\omega_{2,2}R0^2\right)^{\omega_{2,1}}, \tag{S9}$$

Hence, this parameterisation permits the proportion of inter-host transmission contributed by host 1, $R0_{2,1}/(R0_{2,1} + R0_{1,2})$, to be either linearly, for $\omega_{2,1} = 0.5$, or non-linearly, for $\omega_{2,1} > 0.5$, related to $R0$. Note that restricting $\omega_{2,1} \in [0.5, 1]$ ensures that at least 50% of inter-host transmission is attributable to host 1 (i.e., dogs). Substituting Equations S5, S6, S8 and S9 into the $\mathbf{K}$ matrix (Equation S2) yields

$$\mathbf{K} = \begin{bmatrix} \omega_{1,1}R0 & \left(\omega_{1,1}\omega_{2,2}R0^2\right)^{\omega_{1,2}} \\ \left(\omega_{1,1}\omega_{2,2}R0^2\right)^{\omega_{2,1}} & \omega_{2,2}R0 \end{bmatrix} \tag{S10}$$

as written in the main text.

1.1.2 Transmission dynamics

We model the rate of change in the mean number of hookworms in (recipient) host i at time t, $W_i(t)$, using an ordinary differential equation,

$$\frac{dW_i(t)}{dt} = \frac{\mu_L}{\rho} R_{E\to i} L(t) - (\mu_W + \mu_i) W_i(t). \tag{S11}$$

Here, μ_L, μ_W and μ_i are the per capita mortality rates of adult and larval hookworms and hosts respectively, ρ is the female sex ratio (proportion of worms/larvae that are female) and $L(t)$ is the mean number of larvae in the environment. Note the inclusion of ρ because $W_i(t)$ is the average number of female *and* male worms and $R_{E\to i}$ is defined in terms of female worms only. Since larval dynamics operate on a much shorter timescale than adult worm dynamics, it can be assumed that $L(t)$ is at equilibrium with respect to the mean number of adult worms in (donor) host $W_j(t)$. Therefore, we can write

$$L(t) = \frac{\rho}{\mu_L} \sum_j (\mu_W + \mu_j) R_{j \to E} W_j(t) \Omega\big(W_j(t), k_j(t), b\big) \Phi\big(W_j(t), k_j(t)\big), \tag{S12}$$

where $\Omega(\cdot)$ and $\Phi(\cdot)$ are density-dependent processes (see section 1.2) and $k_j(t)$ is a dynamic function (see section 1.3) that describes the overdispersion/aggregation of hookworms among hosts. Substituting Equation S12 into Equation S11 and writing in terms of intra- and inter-host reproduction numbers (Equation S1) yields

$$\frac{dW_i(t)}{dt} = \sum_j (\mu_W + \mu_j) R0_{i,j} W_j(t) \Omega\big(W_j(t), k_j(t), b\big) \Phi\big(W_j(t), k_j(t)\big) - (\mu_W + \mu_i) W_i(t). \tag{S13}$$

Equation S13 can be expressed more succinctly in terms of effective reproduction numbers, $Re_{i,j}$, which are given by adjusting the basic reproduction numbers for the effects of density dependencies (Churcher et al., 2006),

$$Re_{i,j} = R0_{i,j} \Omega\big(W_j(t), k_j(t), b\big) \Phi\big(W_j(t), k_j(t)\big). \tag{S14}$$

Therefore, as given in the main text, Equation S13 can be re-written as

$$\frac{dW_i(t)}{dt} = \sum_j (\mu_W + \mu_j) Re_{i,j} W_j(t) - (\mu_W + \mu_i) W_i(t). \tag{S15}$$

1.2 Density dependencies

The functions $\Omega(\cdot)$ and $\Phi(\cdot)$ in Equations S12, S13 and S14 denote density-dependent fecundity and mating probability functions respectively. Density-dependent fecundity is necessary to constrain the parasite population (Anderson and May, 1991) and $\Omega(\cdot)$ captures the net reduction in fecundity accounting for both $W_j(t)$ and the overdispersion (aggregation) of worms among hosts, $k_j(t)$ (Churcher et al., 2005, 2006). Here, $\Omega(\cdot)$ is derived from a power relationship of the form $y \propto n^b$ between egg output (eggs per gram of faeces, EPG, y) and (total) worm burden, x (Anderson and Schad, 1985; Neves et al., 2021),

$$\Omega\big(W_j(t), k_j(t), b\big) = \sum_{n=1} f\big(n; W_j(t), k_j(t)\big) n^{b-1}, \tag{S16}$$

where b controls the severity of density dependence and $f(\cdot)$ is the probability mass function of a zero truncated negative binomial distribution.

The function $\Phi(\cdot)$ in Equation S12 is the mating probability (May, 1993, 1977) which, assuming a completely polygamous system (i.e., one male can mate with all females in a single host), is given by

$$\Phi\big(W_j(t), k_j(t)\big) = 1 - \left(1 + \frac{\rho W_j(t)}{k_j(t)}\right)^{-(k_j(t)+1)}, \tag{S17}$$

1.3 Overdispersion dynamics

The killing of adult worms in the treated fraction of the host population results in changes to the distribution of adult worms—initially increasing overdispersion/aggregation—as a function of the pre- and post-treatment values of $k_i(t)$ and $W_i(t)$ (Collyer and Anderson, 2021),

$$k_i(t) = \begin{cases} k_i^* & t{=}\tau{-}\delta t \\ \dfrac{k_i^* W_i(t)}{(1+k_i^*)W_i^* - k_i^* W_i(t)} & t{=}\tau{+}\delta t \\ \dfrac{W_i(t)^2\big(W_i^* - W_i(\tau{+}\delta t)\big)^2}{\big(W_i^{*2}/k_i^*\big)\big(W_i(t) - W_i(\tau{+}\delta t)\big)^2 + \big(W_i(\tau{+}\delta t)^2/k_i(\tau{+}\delta t)\big)\big(W_i(t) - W_i^*\big)^2} & t{>}\tau{+}\delta t. \end{cases} \tag{S18}$$

Here, k_i^* and W_i^* denote the overdispersion and mean worm burden just before a treatment, and $k_i(t{+}\delta t)$ and $W_i(t{+}\delta t)$ the corresponding values immediately after a treatment. Note that $k_i(t)$ and $W_i(t)$ only take their endemic equilibrium values immediately prior to the *first* simulated treatment round but for convenience we use the k_i^* and W_i^* notation.

REFERENCES

Anderson RM, Truscott J, Hollingsworth TD. The coverage and frequency of mass drug administration required to eliminate persistent transmission of soil-transmitted helminths. *Philosophical Transactions of the Royal Society B Biological Sciences* **369** (2014) 20130435. doi:10.1098/rstb.2013.0435.

Anderson RM. Helminth infections of humans: Mathematical models, population dynamics, and control (Academic Press), *Advances in Parasitology*, vol. 24 (1985), 1–101. doi:10.1016/S0065-308X(08)60561-8.

Diekmann O, Heesterbeek J, Metz J. On the definition and the computation of the basic reproduction ratio R0 in models for infectious diseases in heterogeneous populations. *Journal of Mathematical Biology* **28** (1990) 365–382. doi:10.1007/BF00178324.

Dobson A. Population dynamics of pathogens with multiple host species. *The American Naturalist* **164** (2004) S64–S78. doi:10.1086/424681.

Fenton A, Streicker DG, Petchey OL, Pedersen AB. Are all hosts created equal? partitioning host species contributions to parasite persistence in multihost communities. *The American Naturalist* **186** (2015) 610–622. doi:10.1086/683173.

Churcher TS, Filipe JAN, Basáñez MG. Density dependence and the control of helminth parasites. *Journal of Animal Ecology* **75** (2006) 1313–1320. doi:10.1111/j.1365-2656.2006.01154.x.

Anderson R, May R. *Infectious Diseases of Humans: Dynamics and Control* (Oxford: Oxford University Press) (1991).

Churcher TS, Ferguson NM, Basáñez MG. Density dependence and overdispersion in the transmission of helminth parasites. *Parasitology* **131** (2005) 121–132. doi:10.1017/S0031182005007341.

Anderson RM, Schad GA. Hookworm burdens and faecal egg counts: an analysis of the biological basis of variation. *Transactions of the Royal Society of Tropical Medicine and Hygiene* **79** (1985) 812–825. doi:10.1016/0035-9203(85)90128-2.

Neves MN, Gower CM, Webster JP, Walker M. Revisiting density-dependent fecundity in schistosomes using sibship reconstruction. *PLoS Neglected Tropical Diseases* **15** (2021) e0009396. doi:110.1371/journal.pntd.0009396.

May RM. Biased sex ratios and parasite mating probabilities. *Parasitology* **107** (1993) 287–295. doi:10.1017/s0031182000079269.

May RM. Togetherness among schistosomes: its effects on the dynamics of the infection. *Mathematical Biosciences* **34** (1977) 301–343. doi:10.1016/0025-5564(77)90030-X.

Collyer BS, Anderson RM. Probability distributions of helminth parasite burdens within the human host population following repeated rounds of mass drug administration and their impact on the transmission breakpoint. *Journal of The Royal Society Interface* **18** (2021) 20210200. doi:10.1098/rsif.2021.0200.
